# Improving Zinc and Iron Biofortification in Wheat through Genomics Approaches

**DOI:** 10.1007/s11033-022-07326-z

**Published:** 2022-06-03

**Authors:** Shabir Hussain Wani, Kiran Gaikwad, Ali Razzaq, Kajal Samantara, Manjeet Kumar, Velu Govindan

**Affiliations:** 1 Mountain Research Centre for Field Crops, Sher-e-Kashmir University of Agricultural Sciences and Technology of Kashmir, 192102 Khudwani, J&K India; 2 ICAR-Indian Agricultural Research Institute, Pusa Campus, 110012 New Delhi, India; 3 Centre of Agricultural Biochemistry and Biotechnology, University of Agriculture Faisalabad, 38040 Faisalabad, Pakistan; 4 Department of Genetics and Plant Breeding, Centurion University of Technology and Management, 761211 Odisha, India; 5 Global Wheat Program International Maize and Wheat Improvement Center Texcoco Mexico, Texcoco, Mexico

**Keywords:** Wheat, Genomics, Gene mapping, iron, Zinc

## Abstract

Globally, about 20% of calories (energy) come from wheat. In some countries, it is more than 70%. More than 2 billion people are at risk for zinc deficiency and even more, people are at risk of iron deficiency, nearly a quarter of all children underage group of 5 are physically and cognitively stunted, and lack of dietary zinc is a major contributing factor. Biofortified wheat with elevated levels of zinc and iron has several potential advantages as a delivery vehicle for micronutrients in the diets of resource-poor consumers who depend on cereal-based diets. The conventional breeding strategies have been successful in the introduction of novel alleles for grain Zn and Fe that led to the release of competitive Zn enriched wheat varieties in South Asia. The major challenge over the next few decades will be to maintain the rates of genetic gains for grain yield along with increased grain Zn/Fe concentration to meet the food and nutritional security challenges. Therefore, to remain competitive, the performance of Zn-enhanced lines/varieties must be equal or superior to that of current non-biofortified elite lines/varieties. Since both yield and Zn content are invisible and quantitatively inherited traits except few intermediate effect QTL regions identified for grain Zn, increased breeding efforts and new approaches are required to combine them at high frequency, ensuring that Zn levels are steadily increased to the required levels across the breeding pipelines. The current review article provides a comprehensive list of genomic regions for enhancing grain Zn and Fe concentrations in wheat including key candidate gene families such *NAS, ZIP, VLT, ZIFL*, and *YSL*. Implementing forward breeding by taking advantage of the rapid cycling trait pipeline approaches would simultaneously introgress high Zn and Fe QTL into the high Zn and normal elite lines, further increasing Zn and Fe concentrations.

## Introduction

Micronutrients deficiency has an influential impact on human diets and is the leading concern for malnutrition globally. In developing nations, the hidden hunger drives severe health concerns and became an urgent economic load on the health care system. This alarming situation is also challenging the agricultural scientific community because of the rising global population and augmenting food demand. Malnutrition resulted mainly due to the deficicencies of vitamin A, zinc, iodine, folate, and iron [1]. Mineral deficiencies, primarily Zinc (Zn) and Iron (Fe), are impacting over half of the world population considering their cereal crop dependency, especially on wheat, rice, and maize for their daily diet. Around 50% of the global cereal cultivated soils are deficient in Zn content while Fe deficiency is substantially reported in arid regions along with high pH and calcareous soils. Hence, bio-fortification of these crop species with microelements can assure a sustainable solution for food security [2]. The trace element Zn is very important as it plays a key role in many enzymes that are involved in the metabolism of auxin and carbohydrates, protein synthesis, and membrane integrity [3, 4]. It also involves in development of pollen, fertilization, and synthesis of chlorophyll. Another important element, Fe takes part in the electron transport chainand cytochrome and also aids inactivation of several enzymes. Malnutrition of micronutrients causes child death causalities every year hence it is mandatory to nourish the human population with these very essential trace elements [3, 4].

Wheat (*Triticum aestivum* L.) being extensively cultivated and consumed cereal staple crop can be exploited as an appropriate target for Zn and Fe bio-fortification for minimizing the nutritional gaps in cereal diet-based nations. The nutrient deficiencies of Zn and Fe in the cereal crops are posing serious challenges to fulfil the nutritive needs of women and children around the globe. The unavailability of these mineral nutrients causes serious sickness in the developing countries. Hence, optimum intake of Zn and Fe is crucial to achieve the goal of food security and overcome the hidden hunger [5]. In wheat these nutrients are readily present in aleurone layer in excess amount, but the bioavailability of Zn and Fe are very low due to the presence of phytate in wheat seeds which is considered as a major factor to chelate metal ions. The phytate is consist of myo-inositol and phytic acid that is the greatest brunt on Zn and Fe uptake in human body. It has the strong properties to bind the free metal ions and chelate them to make as salt compounds. Biofortification of wheat is the promising, cost-effective and most sustainable approach to deal with the Zn and Fe deficiencies. It is a technique to develop biofortified crops in which Zn and Fe are readily available. Biofortification method is more convenient and sustainable as compared to other conventional methods of fortification because there will be no additional cost to make Zn and Fe available for human intake [6]. To cope with this problem, several initiatives have been taken to improve the genetic make up of wheat through conventional and modern breeding approaches. The aim is not only to enhance the nutrition value of wheat but also to improve the bioavailability of Zn and Fe in the edible parts of plant which can only be possible if the anti-nutrient characteristics of phytate is modified. Hence, there is a need to fix this problem in long run by developing wheat genotypes with improved Zn and Fe contents and increased bioavailability of these nutrients through reducing the phytate content in seeds. Wheat bio-fortification can be achieved through new varietal development with inherently elevated Fe and Zn content in their grains. However, the ambiguity of genetic regulators and metabolic pathways links that regulates the Zn homeostasis are still need to be elucidated which makes the nutritional breeding approach quite complicated [7].

Again, the internal mobilization patterns of these trace elements are affected owing to the differences in Zn use efficiency of wheat crops, variable translocation into the grains, and genotype-dependent source-sink relations [8]. The nutritional quality parameters viz., Zn and Fe are inclined to be polygenic. Hence broadening the genetic base through the exploitation of landraces, wild cultivars via advanced breeding strategies would assist in identifying genetic control and its effects. Again, the genetic base dissections including the exploration of loci, controlling important nutritional quality traits can aid in further enhancing the wheat nutritional values [9]. Quantitative trait loci (QTL) studies have successfully uncovered the genetic basis of QTL positions for Zn and Fe concentrations in wheat bi-parental populations but possess low resolution [10–12]. Hence, now genome-wide association studies (GWAS) are extensively used to dissect the genetic bases of complex crop traits [13] and also have widely utilized to scrutinize the genetic regulation of complex traits in wheat. GWAS offers better QTL resolution, broad allele coverage, as compared to traditional QTL mapping. It also can analyze a broad range of natural germplasm resources including landraces, wild relatives, elite cultivars, and advanced breeding lines [9]. In wheat, GWAS has been used in several studies to examine the genetics of improving milling and baking qualities of wheat [14]. This review briefs about the significance of Zn and Fe in human nutrition along with their genetic control in wheat. We have also described the various mechanisms such as metal transporters that facilitate uptake, translocation as well as storage of grain Fe and Zn. Moreover, the genetics and molecular breeding aspects have been addressed in wheat for grain Fe and Zn biofortification.

## Nutritive value of Zinc and Iron

Micronutrients are very much essential to sustain adequate growth and development. These elements play a key role being the structural component or cofactor to multiple enzymes and proteins that are concerned with various biological and physiological activities [15]. There are approximately fifty essential micronutrients are required by the human body to perform all the crucial metabolic functions. Lacking any of these micronutrients may cause adverse affect on human growth and development. At present, there are several complications associated with malnutrition which have become a great matter of concern worldwidely. One of the main reasons for malnutrition is the unavailability of vital micronutrients including Zinc (Zn) and Iron (Fe). These trace elements though required in a minor amount by our body system, but their deficiemcu can cause numerous disorders [14].

Zn deficit has affected around 2.2 billion individuals throughout the globe and has placed at 11th rank among the global threat for mortality and 12th for the burden of disease. According to the country food balance sheet, nearly 15–20% of the world population are unable to fulfil their Zn requirements because of their insufficient absorbable Zn carrying national food supplies [16]. Wessells et al. [16] suggested a cut-off using a plasma zinc concentration of 50 µg/dL (7.65 µmol/L) for harsh zinc deficit in adults. The everyday Zn needs of an adult individual range from 8 to 11 mg per day while pregnant and lactating women require the highest intakes at 11–13 mg per day [17, 18]. In our body, limited dietary intake, scanty absorption, additional loss or use during disease conditions resulted in Zn deficiency [19]. The most common trace element Zn performs a vital role in biochemical processes that affect growth and development by acting as a structural, catalytic, and signaling factor in human body [20] for several proteins that are involved in neuronal development, immunity, signal transduction, cell proliferation, migration, survival, and death [21]. The catalytic enzyme functions or the structural stability of proteins are supported by the direct involvement of Zn for their folding and oligomerizations. In hormones like steroids and thyroid as well as A and D vitamins, the prosthetic group of innumerable enzymes and the receptor proteins are formed by the Zn element. The synthesis and/or degradation of certain metabolic products viz., carbohydrates, lipids, proteins, and nucleic acids are dependent upon various enzymes like oxidoreductases, transferases, hydrolases, lyases, isomerases, and ligases whose have broad Zn fraction [22]. Due to the deficit of Zn content in the body, retarded growth, impaired immune and nervous systems have resulted. The general clinical symptoms in the human body are skin lesion formation, depressing mental functions, retarded night-vision, anorexia, hypogonadism, delayed wound healing including a high incidence of diarrheal, pneumonia, and malaria because of defective immune function. Again, the Zn deficiency at a cellular level as well as its overload can generate oxidative stress. However, the overuse of Zn may also cause toxicity. An appropriate check of cellular zinc is crucial for the balance between health and disease [15].

Like Zn, there is another important trace element called Iron (Fe) is also responsible for enormous body functions. Iron serves as the central hemoglobin atom as well as the constituent of myoglobin [18, 23] whose role is to store oxygen in muscle tissues and of the cytochrome system that is vital in the energy liberating means of cellular respiration. The prime reason for Fe deficiency involves limited bioavailable iron absorption, rapid use of Fe during a massive growth phase, menstrual cycle, pregnancy as well as gastrointestinal blood loss due to hookworm and whipworm [24–27]. Iron deficiency impacts over 2 billion individuals at a global level and iron-deficiency anemia remains the core reason for anemia worldwide [28]. In mitochondria, Fe acts as a cofactor for heme-containing enzymes and non-heme iron-containing enzymes in the electron transport chain. Hence Fe deficiency interferes with the heme and iron-sulfur clusters biosynthesis which eventually disrupts the hemoglobin, myoglobin, nitric oxide synthase, and cytochromes synthesis. And these resulted in fatigue, lethargy, and dyspnea. Fe is also essential for various enzymes that are associated with the replication and repair of DNA and aids in the regulation of the cell cycle. Fe is even crucial for immune cell growth, proliferation, differentiation, and for particular cell-mediated effector pathways [29]. Fe deficiency is also responsible for heart failure, chronic kidney disease, cancer, and inflammatory bowel disease [30]. However excessive Fe consumption causes hemochromatosis and is characterized by the deposit of Fe in the liver leading to fibrosis. The recommended daily iron consumption varies from 12 to 28 mg/day but a high quantity of iron is recommended for 14–50 year age group female than that of same-aged men. During pregnancy high Fe content (30–38 mg/day) is required while during lactation it is required in a lesser amount (Brown et al. 2021). In addition, since 2020, the human being has been troubled with severe acute respiratory syndrome coronavirus COVID-19 hence these important trace elements are very much critical for immune strengthening.

## Genetic mechanism invloving Zn/Fe absorption and translocation in wheat

Different plants have designed excellent sensing and sophisticated genetic regulators to control the complex mechanics involved in Zn/Fe availability. Higher plants used two major processes to absorb Zn/Fe from soil [31, 32] and evolved various homeostatic networks to mitigate the ZN/Fe toxicity in the cell through controlling the nutrient uptake by cells, intracellular compartmentalization, cytosolic efflux and chelating via Zn/Fe binding ligands [33, 34]. Strategy-1 is a reduction-based mechanism, generally adopted by non-gramineous monocotyledon and dicotyledon plants which involved the direct absorption of Zn^2+^ and Fe^2+^ from the rhizosphere of roots [31, 35]. This led to the transfer of Zn^2+^/Fe^2+^ across the cell membrane facilitated by several Fe/Zn transporter such as iron-regulated transporter (IRT) and zinc-regulated transporter (ZRT)-like proteins [36, 37].

**Fig. 1:**
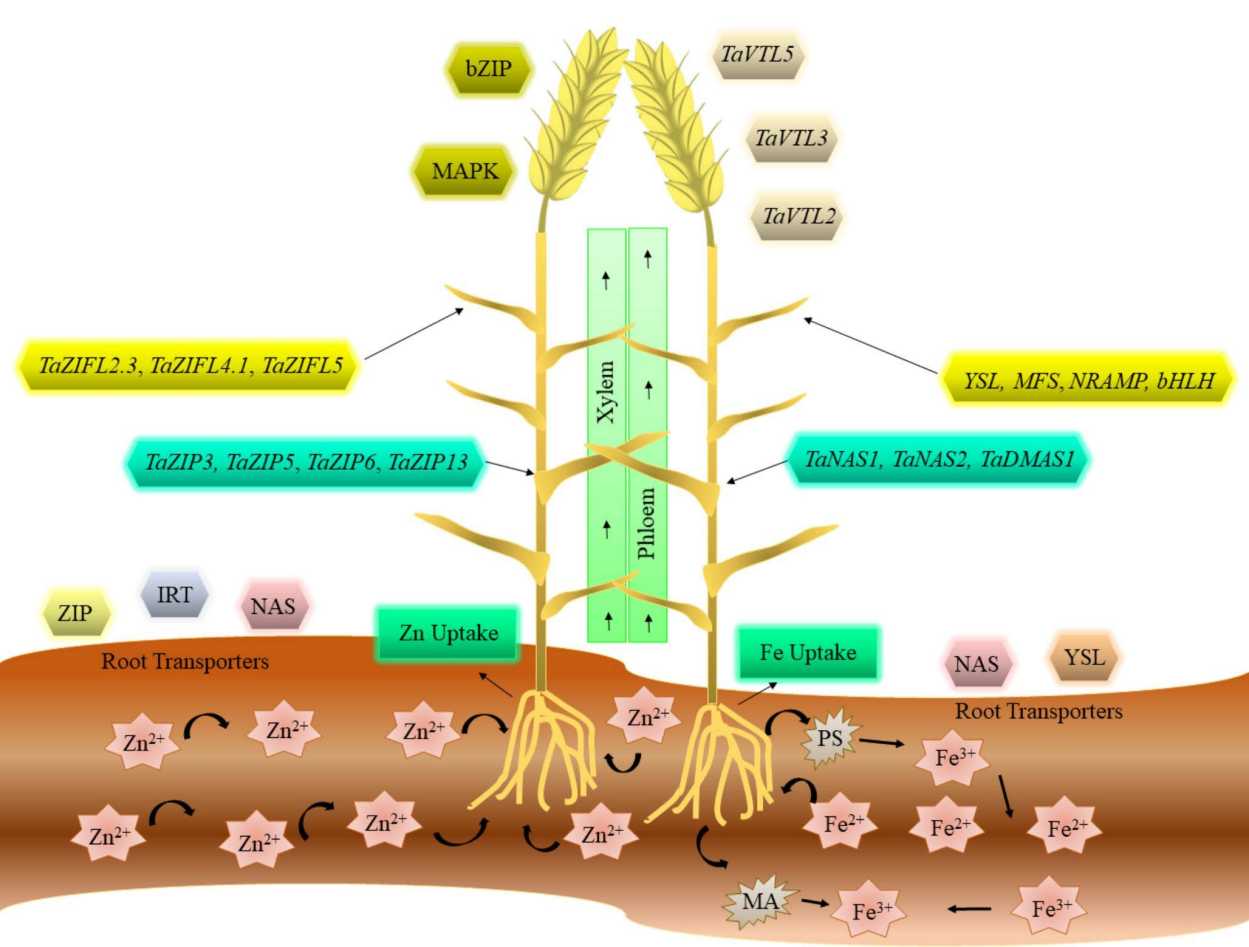
Illustrated the genetic mechanism of Fe/Zn regulating the availability, uptake, and translocation across the different tissues from root to shoot and from shoot to grains in wheat. The roots release the MA and PS which act as a chelating agent to reduce Fe3 + to Fe2 + to make it readily available for root uptake. Different metal transporters (ZIP, IRT-like proteins) assist the Zn2 + absorption in roots while NAS, YSL, and ZIF translocate the Zn/Fe from root to shoot. Some vegetative tissues specific transporters like VTL and MAPK deposited the Zn/Fe in the wheat grains

Most of the gramineous plant species such as wheat adopt the strategy-II (Fig. 1) which is characterized by the release of mugineic acid (MA) by roots and phytosiderophores (PSs) to carry out the Fe^3+^ chelation-based mechanism to chelate Fe^2+^ in soil [38, 39]. Subsequently, yellow stripe-like (YSL) transpoters are vital that are used to transfer the PSs-Fe^2+^ complexes into the root cells and make them readily available for absorption and translocation [40]. A large number of similar types of protein families are participated to transport Zn/Fe at many steps. However, in some cases, Zn and Fe are considered independent metals by plants due to the contribution of multi-genes and complex molecular interactions [41]. For example, different contradictory reports are describing the release of MA by plant roots under Zn/Fe deficiency. A study in wheat showed that the excretion of MA is enhanced under Zn stress while some findings indicated the increase of MA under Fe deficiency [42].

Zn/Fe chelators including nicotianamine (NA) are keys for the sublime flow of Zn and Fe across the root cells and transport the Zn from root to shoot [41, 43]. Methionine is used to synthesize MA in a multistep mechanism which in turn originates from S-adenosyl-methionine in the presence of nicotinamine synthase (NAS) [44]. Many other gene families like ZRT, NRAMP, NAS, YSL, and IRT-like proteins consisting of ZIFL, NAAT, MTP, HMA, and ZIP have also been found to regulate Zn/Fe homeostasis [45, 46]. In the later step, Zn/Fe are shifted into the xylem where Zn can transfer as citrate by forming a complex with organic acids or move as a cation [47], while Fe is chelated [48]. Protein families YSL and ZIP facilitated the movement of Zn/Fe from xylem and phloem to shoot and during grain filling. Due to the discontinuous nature of the xylem in wheat, phloem is the major structure moving all the nutrients into the grain [49]. Followed by Zn/Fe are translocated by forming different complexes with small proteins or NA into the endosperm cavity and embryo aided by metal tolerance protein (MTP) families [50].

Some reports have revealed that most of the nutrients derived from vegetive tissues especially 70% of Zn accumulated at the grain filling stage. The elevated applications nutrients to soil increase the Zn uptake about 3 folds by roots while translocation of Zn enhance up to 8 folds from roots to shoots [51]. However, the nutrient stress conditions hampers the Zn accumulation and its translocation. The development of micro-nutrients dense genotypes and identification of nutrient efficient genotypes is required to overcome micronutrients deficiencies. A better knowledge about stress tolerance signs is needed to for designing satisfactory screening techniques to select micronutrients efficient genotyopes. The micronutrients efficient genotypes has the ability to absorb maximum nutrients through roots and their effieint transportation and translocation from roots to other parts of the tissue under any condition. For example, Gupta et al. [52] identified 28 unique transcripts related to Zn/Fe absorption and transportation mechanisms that involved in efficient transportation and accumulation of Zn and Fe in wheat. Zn stress-tolerant germplasm of wheat utilize particular mechanisms like organic ligands, phytosiderophore, and release of proton to increase Zn absorption efficiency of roots; thus, Zn tolerant genotypes can cultivate on Zn deficient soil [53]. Recently, International Maize and Wheat Improvement Center (CIMMYT) developed a diversity panel through genetic dissection of wheat grain to calculate the Zn concentration. The wheat landraces and wild relatives genomes are recognized to have increased levels of micronutrients in grain. Also, the wild relatives and landraces has very low nutrients needs and can grow under limited supply of nutrients. Introgression breeding was used to transfer some unique traits into modern lines via backcrossing to develop a rich gene pool with enhanced genetic diversity [9, 53, 54]. The results showed that 39 genetic markers were identified controlling the variations in Zn level and detected 7 candidate genes like metal-ion binding and zinc finger motif of transcription-factors.

## Zn/Fe translocation via metal transporters

To maintain Zn/Fe homeostasis, plants established an array of complex regulatory networks to control Zn/Fe uptake, transport, and storage. Despite numerous reports on Zn/Fe deficiency in different plants, these processes still need to be uncovered significantly to present a clear snapshot of genetic regulators involving in translocation in wheat. After uptake by the root rhizosphere, Zn/Fe is translocated to different cells and tissues of the plants according to demand. In the case of Fe, it must be attached with other molecules and transfer as a complex, as Fe^3+^ has low solubility and Fe^2+^ is injurious to plant cells [55]. In wheat, Zn/Fe is deposited in the aleurone layer of the grain which is destroyed during milling. Also, Fe is accumulated in protein storage vacuoles in these tissues that are tightly bound to phytate and became unavailable [50, 56]. Ferritin is considered a good form of bioavailable Fe storage in endosperm amyloplast stored in the form of Fe-rich nanoparticles [57]. Hence, it is very crucial to consider all the Zn/Fe contents in the grains including specialized metal transporters, chelators, and other protein subunits that responsible for its translocation across the plant cells and bioavailability.

The translocation of Zn/Fe from root to shoot and their deposition in grains are the main reasons for Zn/Fe deficiencies in wheat. It is important to understand the underlying pathways for translocation and identified unique metal transporters for efficient biofortification. Several metal transporters have been identified for Zn/Fe translocation in wheat loading [45]. For example, Li et al. [58] reported that *TaZIPs* transporters were involved in the translocation of Zn from root to shoot and maintain ion homeostasis for greater Zn tolerance to wheat. The results showed that the *TaZIP5*, *TaZIP6*, and *TaZIP13* were associated with Zn uptake while several *TaZIP* genes facilitated the Zn translocation. Zn deficiency triggered the expression of transcription factors of group F bZIP and 14 TaZIP transporters in wheat. Functional analysis of Group F TabZIPs revealed the presence of cysteine–histidine-rich motifs, that were supposed to be involved in Zn^2+^ binding under a limited supply of Zn. Zn-deficiency-response elements (ZDREs) were found to operate in ZIPs and some ZIPs including *TaZIP3*, *TaZIP5*, *TaZIP7*, and *TaZIP13*can translocate Zn. Additionally, the conservation of ZDREs differs among ZIPs indicating that each ZIPs can have a unique function in Zn homeostasis [59].

Genetic dissection of pathways controlling the grain Zn concentration was performed to study the putative genes associated with higher accumulation of Zn in wheat grains. Genotyping of European wheat cultivars identified novel genomic regions linked with Zn uptake and translocation. Some candidate genes representing mitogen-activated protein kinase (MAPK) and bZIP were also identified about Zn concentration in wheat grain [60]. Nie et al. [61] discovered that the ample supply of nitrogen improved the Zn concertation and increase the translocation rate from root to shoot in winter wheat grains. The nitrogen supply up regulated the expression of *TaZIP3* and *TaZIP7* genes in different tissues. In another study, the role of ZIPs in the uptake of both Zn/Fe was examined under nutrient deficiency conditions. The Overexpressed genes *TaZIP3* and *TaZIP7* showed higher accumulation and translocation of Zn/Fe in roots and shoots of wheat [62].

Nicotianamine synthase (NAS) is regarded as the largest gene family of NAS genes due to the recent discovery of 21 NAS genes in bread wheat. These genes revealed the complex genomic architecture of Fe homeostasis and may function in several pathways involving Fe uptake and translocation [63]. Beasley et al. [64] characterized deoxymugineic acid synthase (DMAS) and nicotianamine aminotransferase (NAA) genes for Fe uptake and translocation in wheat. Under Fe stress conditions three homeologs genes including *TaNAAT1*, *TaNAAT2*, and *TaDMAS1* were detected that expressed at higher levels in roots. The NAS genes *TaNAS1* and *TaNAS2* were also upregulated in roots and shoots and enhanced the Zn/Fe uptake and transport in winter wheat subjected to increased applications of nitrogen [65]. Numerous genes have been upregulated in different tissues under nutrients starvation in wheat [66, 67].

Likewise, Zinc–Induced Facilitator-Like Family (ZIFL) transporters comprised of many genes that have been studied for their function in translocation of Zn/Fe in wheat [68]. Sharma et al. [69] analyzed the ZIFL genes under Zn/Fe starvation in wheat and identified some genes like *TaZIFL2.3*, *TaZIFL4.1*, *TaZIFL4.2*, *TaZIFL5*, *TaZIFL6.1*, and *TaZIFL6.2* which were up regulated in root in response to nutrient stress. Wang et al. [67] studied the molecular pathways governing the Fe stress response in wheat. The genes involved in the biosynthesis of phytosiderophore were up regulated in roots, showing the significant importance of deoxymugineic acid (DMA) in Fe uptake. The higher expression of *DMAS* and *NAS* genes in flag leaves and grains indicated the role of DMA in Fe chelation and translocation especially during grain filling in wheat. Furthermore, *BASIC HELIX-LOOP-HELIX* (*bHLH*), *NATURAL RESISTANCE ASSOCIATED MACROPHAGE PROTEIN* (*NRAMP*), *YELLOW STRIPE-LIKE* (*YSL*), and *TRANSPORTER OF MUGINEIC ACID* (TOM) were also highly expressed in Fe starvation. Similarly, Wang et al. [70] also studied that Fe deficiency triggered some genes related to *OLIGOPEPTIDE TRANSPORTER* (*OPT*), *ATP-BINDING CASSETTE* (*ABC*) transporter superfamily, *MAJOR FACILITATOR SUPERFAMILY* (*MFS*) family, *NRAMP* family, and *bHLH* family which showed higher accumulation in roots and flag leaves bread wheat. Other metal transporters include the super family of vacuolar iron transporter (VITs) that have been extensively characterized in cereals. A gene *TaVIT2* was investigated for its role in Fe translocation and results showed upregulation of *TaVIT2* in vegetative tissue cause 2 fold increase in Fe accumulation [71]. Sharma et al. [72] revealed the upregulation of certain genes such as *TaVTL2*, *TaVTL3*, and *TaVTL5* in different tissue of wheat and regulate the Fe homeostasis in the cell.

## Biofortification of wheat through conventional breeding

Developing biofortified varieties of food crops through conventional breeding is still a most preferred and cost-effective approach. Major public and private sector organizations in Asia and African countries in collaboration with international organizations like CIMMYT, ICARDA, Harvest Plus are actively involved in developing bio-fortified varieties of Wheat. Collaborative efforts of these organization with national research institutes have yielded many Fe and Zn rich varieties of wheat in India, Pakistan, Nepal and Bangladesh and few other countries. In India, development of Fe and Zn rich wheat varieties has gained a momentum in last few years. A total of 11 varieties of bread wheat and 5 varieties of durum wheat rich in either Fe or Zn or both have been developed by five research institutes and were released by central variety release committee, Government of India. Three wheat varieties viz., WB 02, HPBW 01 a selection form 3rd HPYT (HarvestPlus Yield Trial) by ICAR-Indian Institute of Wheat and Barley Research, Karnal and Punjab Agricultural university, Ludhiana India, respectively share a common parentage (T.DICOCCON CI9309/AE.SQUARROSA (409)/3/MILAN/S87230//BAV92/4/2*MILAN/S87230 //BAV92) [73] and HI 1633 developed by ICAR-Indian Agricultural Research Institute (ICAR-IARI), Indore from an indigenous cross (GW 322 / PBW 498) possess both high Fe and Zn content (Yadava et al. 2020). Three bread wheat varieties are rich in Zn content viz., PBW 757 (42.3 ppm), PBW 771 (41.4 ppm) and HD 3171 developed by ICAR-IARI, New Delhi for North-Eastern Plains of India has highest content of Zn in the grains (47.1 ppm) than any bread wheat variety commercially grown in India [74]. In addition, HUW711 a biofortified Zn wheat variety released by Banaras Hindu University, India. Recently released four bread wheat varieties viz., DBW 187, HI 1605, HD 3249 and HD 3298 have more than 43 ppm Fe content [75]. Durum wheat breeding program of IARI, Indore is leading in developing high Fe and Zn rich varieties. Their durum variety HI 8777 possesses high Zn (43.6 ppm) and Fe (48.7 ppm) content; two more varieties viz., HI 8759, HI 8805 have > 40 ppm Fe and Zn content [75]. One durum wheat variety MACS 4028 having high Fe (46.1 ppm) and Zn (40.3 ppm) developed by Agharkar Research Institute, Pune, India have been included in the United Nations Children’s Fund (UNICEF) to tackle the hidden hunger in the rural areas of India under the National Nutrition Strategy (MACS 4028 https://dst.gov.in/scientists-ari-pune-develop-biofortified-high-protein-wheat-variety). In 2012, the HarvestPlus program of CGIAR, in alliance with Banaras Hindu University, India, and CIMMYT, developed high-Zn genotypes with better agronomic benefits designated as BHU 1, BHU 3 (Akshay), BHU 5, and BHU 6. Through public–private partnership, HarvestPlus has reached more than 500,000 wheat farmers in the Eastern Gangetic Plain of India [76].

Fast track multiplication of quality seed is important for spread of variety on large acreage. Public -private partnership (PPP) is now commercially been exploited in India. Indian seed sector involving smaller to bigger companies and using PPP model for seed availability. The ‘Nirmal seeds’ of India in collaboration with HarvestPlus took up large scale multiplication of seeds of two Zn rich varieties namely Abhay (Zinc Shakthi) and Akshay (BHU 3) over 250 diverse sites in 2015-16. Another medium scale seed company ‘Astha Beej’ also marketed 4 tons of ‘Zinc Shakthi’ seeds in 2014 as “Chitra” and also multiplied its seeds in over 100 acres for marketing in 2015 [76]. This has helped in quicker availability of seeds of Zn rich varieties to the farmers of Indo-Gangetic plains.

In other Asian countries like Pakistan, a Zn rich variety ‘Zincol 2016’ in the background of wheat variety NARC 2011 was released in 2016 in collaboration with HarvestPlus and CIMMYT. Another Zn rich variety ‘Akbar 2019’ released by the Ayub Agricultural Research Institute (AARI) in Faisalabad, Punjab during 2019 was grown by farmers in Pakistan during the 2020–2021 planting season. In addition, another Zn biofortified wheat variety ‘Nawab 21’ released in Pakistan. The country has a plan to produce more than 4000 tons of seed of the two released zinc wheat varieties in 2020-21 crop season (HarvestPlus 2020 https://www.harvestplus.org/knowledge-market/in-the-news/pakistan-farmers-grow-new-zinc-wheat-variety-improved-nutrition).

Furthermore, human intervention trials to determine the bioavailability of the zinc in the biofortified lines are currently being carried out in Pakistan [77] In Bangladesh, the Bangladesh Agricultural Research Institute (BARI), in collaboration with CIMMYT, has developed blast resistant, Zn enriched wheat variety ‘BARI Gom 33’ for commercial cultivation in 2017 [78]. These biofortified varieties are extremely important in South Asian countries like India, Pakistan and Bangladesh where people do not consume enough Fe and Zn in their diets. Involvement of these biofortified varieties in Public Distribution System where food grains are distributed at affordable prices to low-income group of the society will contribute to alleviating hidden hunger in these countries.

## Innovations in molecular breeding tools to develop Zn/Fe biofortified wheat

Genetic mapping approaches like QTL mapping using bi-parental populations and Genome-Wide Association Studies (GWAS) can identify novel genomic regions and genes affecting important nutritional traits in wheat. The genomic resources like high throughput SNP genotyping and the reference genome sequences will accelerate the mapping of loci for iron and zinc content in wheat. This will facilitate the development and deployment of molecular markers into an elite genetic background and will increase the efficiency of bio-fortification breeding programs.

### Quantitative trait loci (QTL) mapping

A good number of studies have been conducted for mapping QTLs of grain Fe and Zn content in wheat. Several bi-parental mapping populations have been utilized in mapping these two traits. In majority, these bi-parental mapping populations was developed using bread wheat cultivars ‘as parents, however, related hexaploid species like *Triticum spelta* and stabilized synthetic hexaploid wheat (SHW) lines and tetraploid parents were also utilized for the same. Most of these studies involve MET (multi-environment trial) data for finding consistent QTLs, as grain Fe and Zn contents showed strong genotype x environment interaction.

Several QTLs have been identified for grain Fe and Zn content in wheat which can be deployed in elite genetic backgrounds for enhancing micronutrient contents (Table 1). In one of the important studies conducted at CIMMYT, Mexico, several QTLs have been identified for grain Fe and Zn content in three RIL populations [10, 79]. In a mapping population derived from a cross Seri M82 and SHW line CWI76364, two major QTLs v*iz. QZn.Y13-14_4BS* and *QZn.Across_4BS* explaining the good level of phenotypic variance (PVE) of 19.6% and 17.3% respectively for grain zinc content and QTLs viz., *QFe.Across_7DS* and *QFe.Y13-14_4BS* explaining PVE of 14.5% and 12% respectively for grain Fe content have been identified [79]. Two QTLs on chromosome 4B for Fe and Zn content were pleiotropic indicating their feasibility of simultaneous improvement. In another study by the same group of researchers, two major effect QTLs viz., *QGZn.cimmyt-7B_1P2* on chromosome 7B and*QGFe.cimmyt-4A_P2* on chromosome 4 A explaining largest PVE 32.7% for grain Zn and 21.14% for Fe respectively in two different RIL population have been identified [10]. In another study, conducted at two locations in Turkey and CIMMYT, Obregon, Mexico, for two years, two major effects of stable QTLs for Zn content on chromosome 1D and 6B in tetraploid and hexaploid RIL populations have been reported [12]. The same study [18] was also conducted in glass house in Zn depleted soils with high pH (8.0), a major QTL *QshootZn.sar_1B* with phenotypic variance more than 14% for shoot Zn content was reported. These genomic regions could be important for marker development and their utilization in MAS for improving grain Fe and Zn content. In India, field experiment with plot size of 4.5 m^2^ conducted at two locations for two years from 2011 to 2013, in a DH population derived from a cross Berkut/Kirchauff, three highly stable major QTLs for grain Zn and Fe were reported. The QTL on chromosome 1B and 2B for Zn content explained up to 23.1 and 35.9% PVE, whereas, up to 22.2% PVE was explained by the Fe QTL co-located with the Zn QTL on chromosome 2B [80]. In a RIL population developed using Indian Mega variety PBW 343 and Kenya Swara, Hao et al. [11] identified two novel, stable and large effect QTLs on chromosomes 2Bc and 3AL that explained up to 15% PVE. Many other studies have also identified QTLs for grain Fe and Zn content in wheat [52, 74]. Recently, at CIMMYT, Mexico, two studies have reported major effect QTLs of grain Zn and Fe content. In the first study, a Mexican commercial bread wheat cultivar (Roelfs F2007) was crossed with a high grain zinc content line of Chinese origin to develop a RIL mapping population to identify QTLs. Two QTLs viz., *QGZn.co-5 A for* Zn content and *QGFe.co-3B.1* for Fe content explained more than 14% PVE [81]. They have also identified 55 candidate genes from the markers for grain Zn and Fe content using annotated wheat genome sequence. In the second study, a major effect QTL *QGZn_Y17_6a* on chromosome 6 A with 10.76% PVE was reported for grain Zn content [82]. A RILpopulation developed from a cross Kachu / Zinc shakti was utilized by few studied aiming to identify grian Fe and Zn contents in mature grains (111,113). In a recent study by Rathan et al. [113], three major QTLs with good magnitude of phenotypic variance for grain Fe and Zn content was identified (Table 1).

**Table 1:** Recently reported major effect QTLs of grain Fe and Zn content in wheat

Trait	*QTL*	Flanking markers	Mapping Population	Chromosome No.	LOD	PVE (%)	Additive effect	Developmental stage studied	Agronomic conditions	References
Zn	*QGZn.ada-1B*	rPt-6561	RILs (Adana99 / 70,711) Hexaploid population	1B	3.7	12	5.07	Mature grains	Field trial in 2012-13 at Turkey (Sakarya and Kahramanmaras) and Obregon, Mexico Design: RBD Plot size: Paired row of 1mt lenght Replications: 2	[12]
	*QGZn.ada-1D*	wPt-6979–wPt-730,718		1D	4.2	31	-4.2			
	*QGZn.ada-3 A*	wPt-2698–wPt-0398		3 A	3.8	14	-2.81			
	*QGZn.ada-6B*	wPt-667,798–wPt-7065		6B	7.8	27	-3.54			
	*QGZn.ada-7 A*	wPt-2083–wPt-6083		7 A	3.1	15	-2.48			
	*QGZn.ada-7B*	wPt-733,112		7B	6.6	25	13.1			
Fe	*QGFe.ada-2B*	wPt-9812		2B	5.9	17	3.17			
	*QGFe.ada-2B*	wPt-1394–wPt-7864		2B	5.3	17	2.9			
	*QGFe.ada-6B*	wPt-667,798–wPt-7065		6B	3.9	14	-2.14			
	*QGFe.ada-7B*	wPt-5922		7B	5.6	18	5.78			
Zn	*QGZn.sar-6B*	wPt-743,099- wPt-5037	RILs Saricanak98 / MM5/4 Tetraploid population	6B	3.1	11.7	-9.8	Mature grains	Green house screening in Zn deficient soil with high pH (8.0)	[12]
	*QGZn.sar-1B*	wPt-6434- wPt-1403		1B	2.5	10.6	4.8			
Fe	*QGFe.sar-3 A/3B*	wPt-0784- wPt-8875		3 A/3B	3.5	12.1	-3.2			
	*QGFe.sar-5B*_*TKM*_	wPt-81.25 wPt-9504		5B	4	14.9	2.7			
	*QGFe.sar-5B*_*MCO*_	wPt-7400 wPt-8449		5B	4.7	16.9	4			
Fe	QshootZn.sar_1B	wPt-664,836 wPt-1637		1B	3.5	14.6	-4.7	Shoot		
Zn	*QZn.bhu-1B*	wmc036c–cfa2129	DH (Berkut / Krichauff)	1B	5.0	23.1	0.4	Mature grains	Field experiment at Banaras Hindu University (BHU), Varanasi and Jamalpur, Mirzapur from 2011–2013 Design: RBD Plot size: 4.5 m^2^ Replications: 2	[80]
	*QZn.bhu-2B*	gwm120–wpt2430		2B	3.0	35.9	0.4			
Fe	*QFe.bhu-2B*	gwm120–wpt2430		2B	8.5	22.2	0.4			
Zn	*QZn.Y12-13_4BS*	TP73864-TP71929	RILs (Seri M82 × SHW CWI76364)	4BS	4.04	11.7	1.33	Mature grains	Field trials at CIMMYT Ciudad, Obregon, Mexico form 2014–2014 Design: Augmented Block Design Plot size: 0.1 m^2^ hill plots	[79]
	*QZn.Y13-14_4BS*	TP91631-TP81797		4BS	6.97	19.6	3.47			
	*QZn.Across_4BS*	TP91631-TP81797		4BS	6.64	17.3	2.7			
Fe	*QFe.Y13-14_5BS*	TP91631-TP81797		5BS	5.26	12	0.94			
	*QFe.Across_4BS*	TP91631-TP81797		4BS	5.08	10.7	1.03			
	*QFe.Across_7DS*	TP43715-TP37547		7DS	6.58	14.5	1.14			
Fe	*QGFe.iari-2 A*	gwm359-Xgwm249	RILs (WH542/ SHW line)	2 A	4.1	6.8	--	Mature grains	Field trails at Indian Agriculture Research Institute (IARI), New Delhi, GBPUAT, Pantnagar, and IARI, RS, Pusa Bihar Design: RBD Plot size: 1.25 m^2^ Replications: 2	[84]
	*QGFe.iari-7B*	Xgwm577-Xbarc264		2 A	3.4	6.0	--			
Zn	*QGZn.iari-2 A*	Xgwm359-Xwmc407		2 A	13.5	11.1	--			
	*QGZn.iari-2 A*	Xgwm359-Xgwm249		2 A	11.8	14.4	--			
Fe	*QFe-3D*	Xgwm1047-Xgwm383	RILs (Tabassi/Taifun)	3D	2.76	44.71	-10.64	Mature grains	Field trial at University of Natural Resources and Life Sciences, Vienna in 2004-05	[86]
	*QFe-4D*	Xgwm4670-Xgwm194		4D	2.54	44.6	-10.66			
	*QFe-7B*	Xgwm767-Xgwm3036		7B	2.52	47	10.92			
Zn	*QZn-1 A*	Xgwm3094-Xgwm164		1 A	2.97	50.79	-7.11			
	*QZn-4 A*	Xgwm4026-Xgwm1081		4 A	2.67	40.22	6.28			
Zn	*QGzncpk.cimmyt-1BS*	wPt-3103	RILs (PBW343/Kenya Swara)	1BS	7	11	-2.47	Mature grains	Field trials at CIMMYT Ciudad, Obregon, Mexico in 2012-13 Design: RBD Plot size: Paired row of 1mt lenght Replications: 2	[11]
	*QGzncpk.cimmyt-2Bc*	wPt-6174		2B	6.6	10	2.09			
	*QGzncpk.cimmyt-3AL*	wPt-0286		3AL	9	15	-2.56			
Fe	*QFe-2B*	wPt-7004-wPt-4210	RILs (SHW-L1/Chuanmai 32)	2B	4.6	9.5	4.9	Mature grains	Field experiment at Sichuan Province, China in 2009-10 and 2011-12 Plot size: 2.25 m^2^	[85]
Zn	*QZn-2D*	wPt-730,057-wPt-671,700		2D	5.0	8.6	3.5			
Fe	*QFe-4D*	Xgwm154-Xbarc108	RILs (Chuanmai 42/Chuannong 16)	4D	5.0	19.1	-7.1			
Zn	*QZn-4D*	Xcfa2149-Xbarc48		4D	3.3	15.9	2.5			
	*QZn-3D*	Xbarc6-Xcfe172		3D	4.0	14.5	2.2			
Zn	*QZn.bhu-2B*	989,092|F|0 1,101,425|F|0	RILs (*T. spelta*accession H + 26 (PI348449) /*T.aestivum* cv. HUW 234)	2B	4.81	16.46	2.01	Mature grains	Field trials at BHU, Varanasi, Rajiv Gandhi South Campus, Mirzapur and IARI, New Delhi from 2010–2012 Design: RBD Plot size: 1.2 m^2^ Replications: 2	[87]
	*QZn.bhu-6 A*	998,265|F|0 3,026,160|F|0		6 A	2.61	6.99	1.29			
	*QZn.bhu-6B*	1,001,916|F|0 1,129,916|F|0		6B	3.41	9.7	1.68			
Fe	*QFe.bhu-3B*	3,022,954|F|0 1,102,324|F|0		3B	13.3	25.95	1.63			
	*QFe.bhu-1 A.3*	1,708,014|F|0 1,000,008|F|0		1 A	9.09	16.55	1.35			
	*QFe.bhu-1 A.2*	2,289,695|F|0 1,218,555|F|0		1 A	4.4	7.4	0.83			
Zn	*QGZn.cimmyt 1B_P1*	3,934,172; 3,934,936	RILs (Bubo × Turtur)	1B	8.30	15.10	0.531	Mature grains	Field trials at CIMMYT Ciudad, Obregon, Mexico from 2013-16 Design: RBD Plot size: Paired row of 1mt lenght Replications: 2	[10]
	*QGZn.cimmyt-7B_1P1*	3,945,822; 1132640F0-5CG		7B	7.12	16.75	0.424			
Fe	*QGFe.cimmyt-3A_1P1*	1,234,521; 3034169F0-11AG		3 A	5.26	10.35	-0.139			
Zn	*QGZn.cimmyt-7B_1P2*	1,079,651; 1,262,636	RILs (Louries × Batelur)	7B	20.76	32.79	−1.290			
	*QGZn.cimmyt-1B_1P2*	4,991,478; 3,937,490		1B	8.58	11.25	0.814			
Fe	*QGFe.cimmyt-2A_P2*	4,262,668; 1,226,245		2 A	6.36	14.23	0.112			
	*QGFe.cimmyt-4A_P2*	338,535; 1,211,533		4 A	9.65	21.14	−0.161			
	*QGFe.cimmyt-4D_P2*	2,363,822; 3,961,236		4D	6.45	14.62	−0.109			
Zn	*QGzncpr.cmt-1B* ^*c*^	4,663,991–wPt-10,518	RILs Low Zn/High Zn lines	1B	8.8	15	-2.02	Mature grains	Field trials at CIMMYT Ciudad, Obregon, Mexico in 2012-13 Design: RBD Plot size: Paired row of 1mt lenght Replications: 2	[9]
	*QGzncpr.cmt-5B*	wPt-8163– 1,139,328		5B	6.5	11	1.75			
	*QGzncpr.cmt-6 A*	4,990,410		6 A	4.7	8	1.36			
Zn	*QGZn.co-5 A*	1,244,217 1,272,027|F|0	RILs (Roelfs F2007/ Chinese Parental Line)	5 A	2.69	14.22	1.73	Mature grains	Field trials at CIMMYT Ciudad, Obregon, Mexico from 2016–2018 Design: RBD Plot size: Paired row of 1mt lenght Replications: 2	[81]
	*QGZn.co-7 A*	5,356,706 5,325,178|F|0		7 A	5.47	7.83	1.81			
Fe	*QGFe.co-3B.1*	1,089,107 1,127,875|F|0		3B	3.65	14.56	-1.71			
	*QGFe.co-5 A.2*	1,102,433 988,523		5 A	3.09	6.94	2.09			
Zn	*QGZn_Y17_6a*	1,092,057; 1,082,014	RILs (Kachu / Zinc shakti)	6 A	12.45	10.76	-1.19	Mature grains	Field trials at CIMMYT Ciudad, Obregon, Mexico	[99]
	*QGZn_Y17_1b*	13,142,877; 3,954,275		1B	10.14	9.09	2.16			
Fe	*QGFe_Y17_6b*	1,864,870; 2,278,502		6B	6.03	8.65	0.80			
	*QGFe_Y17_4a*	1,099,697; 5,324,893		4 A	3.93	7.78	0.76			
Zn	QZnC-7D.1	100,024,878–5,050,443	RILs (Kachu / Zinc shakti)	7D	13.67	8.1	0.96	Mature grains	Field trials at CIMMYT Ciudad, Obregon, Mexico from 2017–2020 Design: RBD Plot size: Paired row of 1mt lenght Replications: 2	[111]
	QZnC-1B.1	1,132,017–4,909,722		1B	13.67	7.7	0.98			
	QZnC-2 A.2	1,111,617–982,253		2 A	13.89	6.1	0.78			
Fe	QFeC-2 A.2	1,074,973–2,253,877		2 A	10.2	10.1	-0.22			
	QFeC-6B.1	1,214,987–2,278,502		6B	10.43	10.2	-0.23			
	QFeC-1D.3	981,077–1,167,672		1D	9.52	7.3	-0.18			

Modern wheat varieties/ elite lines have limited variation for grain Zn and Fe, hence utilization of related species, landraces, local cultivars, and SHW lines could serve as important genetic resources for improving grain micro-nutrient content in wheat. The SHW lines harboring useful and novel genetic diversity from the D genome are rich source of genetic variation for high Fe, Zn, and other micro-nutrients [83]. Therefore, their large-scale utilization in breeding programs for developing nutrient-rich elite lines should be on priority. In a RIL population derived from an Indian variety ‘WH 542’ and SHW line, four QTLs each were identified for grain Zn and Fe content [84]. They have also identified a common region in the interval of SSR markers *Xgwm359-Xwmc407* on chromosome 2 A associated with Fe, Zn content. SHW lines were also utilized in developing the RIL population for identification of QTLs for grain Fe, Zn, and other three micro-nutrient content [85]. They have identified 39 QTLs for five micronutrient concentrations and of these, 22 alleles were from synthetic wheat. Similarly, in a bi-parental mapping study, a RIL population derived from an Iranian germplasm line ‘Tabassi’ revealed very high PVE (29.1% and 51.4%) QTLs for grain Fe and Zn content [86]. Another mapping study involving RILs derived from low Zn parent and SHW derived line having high Zn content, showed two major QTLs viz., *QGzncpr.cmt-1Bc, QGzncpr.cmt-5B* on chromosomes 1B, and 5B respectively. The QTL on 1B from high Zn parent explains about 15% of phenotypic variation [9]. Hexaploid species *Triticum spelta* has been utilized in developing the RIL population for mapping QTL of Fe and Zn [87]. The accession of *T. spelta* (H + 26) used in this study was reported to have higher Fe and Zn accumulation capacity. They have reported five QTLs each of Fe and Zn. Two QTLs *viz., QZn.bhu-2B* and *QFe.bhu-3B* for Zn and Fe content explained 16.46% and 25.96% PVE. These stable QTL linked with SNP marker can be effectively used in breeding programs.

Developing countries in Asia and Africa are worst affected by Fe and Zn deficiency. Bio-fortification of wheat varieties by introgression of major effect QTLs of grain Fe and Zn content is the most cost-effective and sustainable approach. To achieve the target level of Fe and Zn, screening of unexplored landraces, related species, wild relatives, promising SHW lines, and alleles from high-nutrient genetic resources are required. Evaluation of mapping populations across the environments for a clear understanding of genotype x environment interaction will be key for identifying stable and major effect QTLs for these two traits. The reported QTLs if deployed in elite genetic backgrounds will lead to a new generation of nutrient-rich breeding lines and will provide a sustainable solution to the problem of malnutrition.

### Genome-wide association studies (GWAS)

Genetic mapping of QTLs has been facilitated greatly in understanding the genetic basis of complex polygenic traits like Fe and Zn accumulation in the wheat grains. Using different bi-parental mapping populations derived from cultivated tetraploid, hexaploid, and SHW lines, several QTLs were identified for these two micro-nutrients (Table 2). However, the QTLs identified using bi-parental populations have low resolution and are confined to the genetic variation present in the two parental lines used in developing the respective mapping population. On the other side, genomic regions identified in GWAS have a good level of QTL resolution, allele coverage as it captures the natural variation from germplasm lines like landraces, local cultivars, SHW lines, released varieties, and advanced breeding lines representing diverse gene pool.

**Table 2:** MTAs identified in different mapping panels using GWAS

SN	Association Panel	Size	Location(s) & Environment(s)	Marker system	No. of markers	Trait Determination	MTA Identified		References
							**Fe**	**Zn**	
1	Synthetic Hexaploid Wheat (SHW) Lines	47	Japan (2)	SSR	70	ICP-AES	03	03	[93]
2.	HarvestPlus Association Mapping Pannel (HPAM)	330	Mexico, India (6)	SNP	28,074	EDXRF	--	39	[9]
3.	SHW Lines	123	Turkey (2)	SNP	35,648	ICP-MS	03	13	[94]
4.	European Elite Wheat varieties Sub-panel	369 183	Germany (3)	SNP	15,523 28,710	ICP-OES	-- --	40 161	[60]
5.	Spring Wheat Reference Set (SWRF)	246	India (2)	SNP	17,937	EDXRF	33	94	[100]
6.	European Elite Wheat varieties Sub-panel	369 183	Germany (3)	SNP	15,523 44,233	ICP-OES	41 137	-- --	[92]
7.	*Aegilops tauschii* Panel	167	India (3)	SNP	5249	ICP-OES	05	04	[95]
8.	Chinese Bread Wheat Varieties Panel	207	China (3)	SNP	2,44,508	AFS-3000	--	29	[89]
9.	HPAM	330	Mexico (2)	SNP	28,074	ICP-MS	65	72	[88]
10.	Chinese Wheat mini-core panel	246	China (2)	SSR	545	ICP-OES	--	11	[90]

GWAS is now being utilized in the identification of marker-trait association (MTA) for grain Fe and Zn content in wheat (Table 2). In the cited GWA studies, different association panels have been used comprising of Harvest Plus Association Mapping Panel (HPAM), European elite wheat varieties, CIMMYTs Spring Wheat Reference Set (SWRF), Chinese bread wheat varieties and mini-core panel, SHW lines, and accessions of *Aegilops tauschii*. HPAM panel comprising of 330 diverse lines have been characterized by SNP markers for find MTAs. This panel was evaluated in India and Mexico in 6 environments for Zn content; GWAS revealed 39 significant MTAs and two large-effect QTLs on chromosomes 2 and 7 [9]. The same HPAM panel was genotyped for Fe and Zn content in mature grain and rachis at two developmental stages. GWA studies revealed 72 and 65 MTAs significantly associated with Zn and Fe content [88]. A panel comprising of 207 Chinese bread wheat lines were used to detect the genetic basis of Zn accumulation in wheat. GWAS was performed using a 660 K SNP array and 29 unique loci associated with Zn grain accumulation having 12–25% PVE were identified [89]. Similarly, in a panel of Chinese mini-core collection comprised of 246 bread wheat lines, 16 loci associated with grain Zn content on 11 chromosomes were identified using SSR markers [90]. Two panels representing 369 European elite wheat varieties and 183 genotypes were used in GWAS for grain Zn concentration using 15,523 and 28,710 polymorphic SNP markers respectively [60]. Notably, 40 and 161 MTAs were detected in elite varieties panel and subpanel respectively (Table 2) of which, highly significant MTAs were found to be present on chromosomes 5 A and 3B. QTLs on these genomic regions were also reported in previous studies [10, 91]. The same two panels were also used to determine the genetic architecture of grain Fe concentration. GWAS revealed 41 and 137 significant SNPs in the whole and subpanel, respectively, including significant MTAs on chromosomes 2 A, 3B, and 5 A for Fe concentration [92].

SHW lines are being used for introgression novel genetic variation into elite lines and it is a good source of high grain minerals. Two studies utilized SHW lines for finding significant MTAs for grain Zn and Fe content using SSR [93] and SNP markers [94]. The diploid species *Aegilops tauschii* which is a D genome donor of cultivated wheat and exploited in developing SHW lines is also an attractive genetic resource for improving mineral nutrition in cultivated wheat. In India, a panel of 167 *Ae. tauschii* accessions were phenotypes for four mineral concentrations including Fe and Zn and genotyped with 5249 GBS markers. For grain Fe and Zn concentrations, five and four significant associations were detected, respectively [95]. Overall, these studies have improved the understanding of the genetic architecture of grain Fe and Zn concentrations in wheat. Though these two traits are under polygenetic control and often influenced by soil parameters, their level can be enhanced by pyramiding multiple major effect QTLs in an elite genetic background. Such genomics studies will not only increase the understanding of the genetics of these traits but also, will improve the breeding efficiency of the biofortification program.

## Transgenic approach to enhance Zn/Fe

Genetic engineering is one of the approaches to develop nutrient-enriched varieties of staple food crops. Conventional and molecular breeding approaches have been comprehensively used to develop bio-fortified varieties and understanding the genetic nature of major nutrient traits respectively. Through transgenic technology, a gene of interest can be directly transferred into an elite variety to elevate the level of micronutrients, provided the nutrient should accumulate in the edible part of the crop without adverse effects on plant physiology or development and economic yield [96]. In wheat, very limited work has been done to improve Fe content using genetic engineering. Borg et al. [97] made an effort to improve Fe content in wheat grain by using the wheat ferritin gene (TaFer1-A). Their results resulted in an improvement of Fe content by 50–85% (44.5 µg/g in endosperm) in grain by overexpressing the Ferritin gene. In a more recent study, a *VACUOLAR IRON TRANSPORTER* (*VIT*) gene (TaVIT2), was overexpressed under an endosperm-specific promoter, in the endosperm of wheat grains that resulted in a 2-fold increase in Fe in white flour fractions without an increase in antinutrient phytate content [71]. To date no efforts have been made in developing Zn-rich transgenic wheat lines, however, the QTL responsible for increasing grain protein content (Gpc-B1) is also known to improve Fe and Zn remobilization in wheat [98]. The cloned *Gpc-B1* gene and mechanism regulating Fe and Zn remobilization pathways provided an avenue for their effective manipulation and translations into nutrient-rich wheat varieties.

## Conclusion and future perspectives

Several genetic and QTL mapping experiments have shown that inheritance of grain Zn (and Fe) is governed by small-to-intermediate-effect QTL of additive effects. Although several QTL of moderate effect for grain Zn and Fe have been found in different germplasm sources, the genetic control of the trait appears to be best treated as polygenic.

The additive and additive × additive gene actions for the selected traits will allow the continuous addition of high grain Zn in high-yielding backgrounds by crossing the best elite lines from the current high Zn breeding lineage with the best elite high-yielding lines. Based on the published results, the most promising candidate hotspot regions for increased grain Zn on chromosomes 2B, 3 A, 4B, 5B, 6B, and 7B; and some QTL regions have a pleiotropic effect for grain Fe as well with grain size QTLs. Moreover, 2B and 4B QTL had a pleiotropic effect for the increased thousand-kernel weight (TKW), suggesting that a simultaneous improvement of grain Zn and seed size is possible.

Of the several QTLs, the promising QTL on chromosomes 2, 5, and 7 has the potential to be used in forward breeding. These QTL showed a significant effect for grain Zn when combined inadequate genetic backgrounds. Further progress is possible by accumulating the additive effect QTL dispersed across lines into elite germplasm through marker-assisted breeding. Implementing forward breeding by taking advantage of the rapid cycling trait pipeline approaches would simultaneously introgress high Zn and Fe QTL into the high Zn and normal elite lines, further increasing Zn and Fe concentrations. In addition, the development of breeder’s friendly KASP markers would accelerate the deployment of promising QTL in the elite backgrounds through marker-assisted breeding as well as the validated markers associated with grain Fe and Zn can be included in the genomic prediction models as fixed effects, and the rest of the markers as random effects for the genomic selection-based breeding. These efforts would enable mainstreaming grain Zn and Fe in elite wheat germplasm, eventually large majority of elite wheat lines from CIMMYT will have increased levels of Zn and Fe which will result in more biofortified varieties released in different target countries of South Asia and Africa.

## Data Availability

Data will be available on request.
